# Exploring challenges and innovation in memory assessment services in England and Wales – a national survey and case study approach

**DOI:** 10.1186/s12913-024-11571-2

**Published:** 2024-09-27

**Authors:** Sarah Jane Smith, Claire Surr

**Affiliations:** Centre for Dementia Research, Leeds Beckett University, Leeds, UK

**Keywords:** Dementia, Memory assessment services, Post diagnostic support, Workforce, Commissioning

## Abstract

**Background:**

To explore the design, delivery models and identify good and innovative practices in Memory Assessment Services (MAS) in England and Wales.

**Methods:**

A two-stage service evaluation comprising (1) on-line survey of MAS providers to identify features of the commissioning models, service design, delivery, and challenges alongside examples of good/innovative practice; (2) qualitative case studies using video/telephone interviews with key staff and people who had used the service.

**Results:**

The 49 respondents to the survey reported a shift in delivery of MAS post COVID and identified key areas for improvement, including a need for specialist staff, support for MCI and rarer dementias, and capacity for post diagnostic support. The 15 case studies illustrated good practice and innovation focusing on post diagnostic support, equity of access, working with external services/service location, MCI and rarer dementia and involving specialist staff.

**Conclusions:**

The evaluation speaks to the importance of (re)evaluation of services to identify local need and the importance of commissioning based on local need and innovative approaches that my sit outside of ‘typical’ MAS pathways.

## Background

Timely dementia diagnosis enables people to receive early treatment and support that brings benefits for the planning of future care, access to dementia medications, better symptom management, implementation of co-ordinated care, postponement of the need for institutional care and greater support for carers [1, 2]. There is also evidence to suggest receiving a timely diagnosis and post-diagnostic support can improve quality of life [3, 4]. Access to a timely dementia diagnosis is a global priority with the WHO global action plan on dementia [5] setting a target of at least 50% of countries having at least 50% of the estimated people with dementia formally diagnosed by 2025. Given an estimated 57.4 million people worldwide were living with dementia in 2019, a number set to triple by 2050 [6], it is important to establish effective, person centred and timely routes and services for diagnosis.

In England timely diagnosis of dementia is driven by policy [7], and dementia-specific clinical guidelines [8] and pathways [9, 10] and memory assessment services (MASs) are the primary means for gaining a dementia diagnosis. Diagnostic procedure and services offered is guided by National Institute of Clinical Excellence (NICE) [8] guidance. This guidance, aimed at practitioners and commissioners, covers diagnosing and managing dementia and includes recommendations on: the process and best practice concerning assessment and diagnosis, psychosocial and pharmacological interventions and managing non-cognitive symptoms. In line with advances in our ability to offer earlier and offer etiological diagnosis [7] clinics increasingly also offer biomarkers and generic testing alongside neuropsychological evaluation to inform diagnosis and post diagnostic support.

MASs were first established in the UK in the 1980s with numbers growing rapidly in the 1990s and 2000s as a means to enhance diagnostic rates in response to national policy and initiatives [11]. As in the UK, the diagnosis and management of people with cognitive impairment typically takes place in memory clinics in most high-income countries (HIC) [12]. For example, in Australia, although there is variation across jurisdictions, typically diagnosis occurs in an outpatient service memory clinic staffed by specialists [13]. Similarly, to the UK, although diagnosis does not occur in Primary Care, research has indicated that the referring GP is an integral part of the diagnostic pathway informing and influencing patient experience and outcomes [13].

Although there are relatively well-established routes to diagnosis in HIC, more than two thirds of people living with dementia are in low and middle income countries (LMIC) [14]. Lower diagnostic rates in LMIC have been attributed to perceptions of dementia being part of the normal aging process, as well as stigmatising views about the condition – hence an avoidance of diagnostic labels [12, 15]. These barriers are often coupled with a lack of diagnostic and post-diagnostic services and resources. Furthermore, the diagnostic standards and criteria (e.g. DSM V) applied in HIC can be difficult to apply in LMIC as neuropsychological tests and diagnostic criteria rely on population norms that are not available in many LMICs [12]. However, as awareness about the importance and value of diagnosis increases so do evidence-based applications of services and tools to enhance diagnosis rates in LMIC countries. For example, in 2016 the World Alzheimer Report advocated the use of community mental health workers as a means to increase diagnosis rates in LMIC [16]. Researchers have also explored the utility and applicably of cognitive assessment tools in LMIC finding that, subject to further research and validation, simple digital tools used in HIC could be useful for the detection of dementia in LMIC [12]. Thus, investigations of practice in HIC, may prove useful for informing future practice in LMICs.

There are approximately 330 MASs in England and a further 22 in Wales. MASs are primarily based in NHS Trusts or primary care organisations, follow a range of models of service design and are commissioned to meet local needs [17]. In England, there is also an optional accreditation route for services. The Memory Services National Accreditation Programme (MSNAP) was established in 2009 to support local service improvement of memory services in the UK. MSNAP developed a set of a quality standards for memory services which are underpinned by research, best practice guidance and legislation. Memory services participate in MSNAP to achieve accreditation against these standards, however in 2021–2023 only 71 services were accredited or working toward accreditation [18]. This is not necessarily reflective of poor-quality MAS in England, but of the optional nature of accreditation and limited incentives for services to become accredited. Thus, there is a limited picture of what MAS services look like across England.

Although a dedicated clinic is the preferred model of care in England, despite attempts to standardised approaches [19], the design and service provision of MAS in England remains difficult to determine with services commissioned and operating in different ways [20]. A study of MASs in England [21] attempted to develop a MAS typology according to characteristics like structure and processes of care but found that variation meant this was not possible. There is greater consistency in MAS provision in Wales, thought to be driven by the way services are commissioned, with 86% of memory clinics provided as part of a wider service such as Community Mental Health Teams for Older People, as opposed to stand-alone clinics. However, variations in post-diagnostic support are still evident [22].

Variability in service design has the benefit of enabling services to be commissioned based on local need but can mean patients may have different services available to them based on postcode. The impact of MAS service structure and on patient outcomes remains unclear [23]. Attempts to address lack of consistency in England include development of quality standards through the Royal College of Psychiatrists, although currently less than 20% of MAS have sought and are accredited through a voluntary process in England [20].

Reports of evidence-based service improvement in MAS in the UK remain limited A regional audit in London found service variation across a range of areas of practice [24], with findings subsequently used to deliver four service improvement projects targeting aspects of service variation and promoting best practice. However, the COVID-19 pandemic had a significant impact on MAS operations with the majority of services closed for a period of months, and on reopening having to quickly establish new ways of remote working, including establishment of guidance to underpin this [25]. Whilst remote assessment and diagnostic methods offer a range of potential benefits in certain circumstances [26], they are not suitable or accessible for all [27].

The available literature on MAS in England and Wales indicates that services are diverse in their provision, there are recognised gaps in service provision and a limited evidence-base to inform practice. COVID-19 has impacted service delivery and necessitated a change in delivery methods. The recent return to usual ways of working provides MAS with a unique opportunity to consider service models and make decisions about longer terms plans for service design, delivery, and improvement. Services need a contemporary evidence-base to do this. This mixed methods study sought to address this need and provide, via a national survey, an overview of the nature of MAS in England and Wales including service models, the impact of COVID-19, the challenges that MAS are facing, perceived gaps in current provision. Using a case study approach we provide examples of innovative service delivery which could be used to inform national and international service development.

## Methods

A mixed-methods service evaluation [28] underpinned by a positive deviance approach [29] comprised of (1) a national survey and subsequent (2) multiple case studies [30] selected from survey respondents.

### Ethics

Ethical approval for the service evaluation was obtained from the Research Ethics Committee at Leeds Beckett University on 21.9.21 (survey) and 29.11.21 (case studies), with the survey approved as an audit and the case studies as service evaluation under the Health Research Authority definitions [31]. The service evaluation included human participants and informed consent was obtain and all procedures carried out in accordance with the Declaration of Helsinki and approved by the aforementioned research ethics committee.

### National survey

A survey of MAS was conducted to (1) provide an overview of the range of service delivery models operating (2) identify areas of need/concern (3) identify innovative practice to inform case study selection (part 2).

### Survey design

The survey design was informed by an expert steering group and public advisory group. The survey included two parts: Part 1 - service model, commissioning, staffing and services. Part 2 - innovative practice across 13 areas of practice interest including examples. The areas of practice interest were identified by advisory groups, drawn from a guide to continual improvement in MAS [32]. Respondents were asked to indicate in the survey if they would be happy to be approached to participate as a case study site.

The survey was completed electronically and distributed via e-mail by the NHS England Clinical Networks and Improvement Cymru to staff who were on their e-mails lists as associated with MAS and was promoted via social media and webinar/conference presentations where MAS staff attended. Due to the way the survey was distributed it is not possible to know the numbers of MAS staff or services reached and thus response rates. Given the primary purpose of the survey was to describe a variety of service models and identify common practice challenges and good practices with MAS, the underpinning sampling approach was thus positive deviance [29]. Therefore, it was not our aim to recruit a representative sample. The survey opened in September 2021 and closed in January 2022.

### Multiple case studies

We adopted a multiple case study approach following Stake’s [33] approach to case study methodology. The quintain or phenomenon of study was how MAS in the UK have innovated their practice to meet local needs in the context of current commissioning models. We adopted the case site selection criteria of particularisation [33] where the power of each site comes from its representation of the local situation rather than its generalisability. This was underpinned by a positive deviance approach, where cases are selected for their ability to provide potential solutions to common problems through approaches already being practised in the community [29]. Our underpinning philosophical stance was pragmatism where applied knowledge is seen as constructed and interpreted based on its application to help solve particular problems [34].

Case study sites in England and Wales were identified based on responses to part two of the survey. Respondents identified and reported on their own interpretation of innovative practice in the survey. Clear indication was provided in the survey as to what elements and types of practice considered good and/or innovative. These topics were based on previously published good practice guide, developed by the authors, an expert consensus group and published with NHS England [32]. For site selection the following criteria were applied:

- Demonstrates at least one example of good or innovative local practice.
- Delivers an example of good practice that is judged by advisory group members to be strong, significant, innovative and potentially replicable in other situations.
- Represents a range of geographic locations/regions.
- Represents a range of MAS commissioning/delivery models.

Anonymised survey data were discussed with the public and expert steering groups where consensus was reached sites were approached to take part.

### Case study data collection and analysis

At each site agreement to take part as a site was obtained from the initial respondent who took responsibility for the identification of participants at each site which comprised two groups (1) Staff who had worked in the service for at least four-months (2) Service recipients and/or their relative/carer who had accessed the MAS in the last two-years.

One-to-one or small group interviews were conducted remotely via Microsoft Teams, or telephone. Interviews took place between March and November 2022. Interviews were conducted by research team comprising the two authors (CS/SS), and two researchers (LG/SB). Each case study site had a team member assigned as the lead for the site (CS/SS/LG or SB) coordinating all communication and data collection and conducting the majority of interviews – a small number of sites had interviews conducted by two researchers due to workloads and timing of interviews to meet practitioner availability. Where appropriate documentary and other secondary evidence was also gathered. The interviews were semi-structured, conversational in style and focused on drawing out key information about the area(s) of good practice and how these had been achieved. All interviews were audio recorded or video recorded on Microsoft teams and later transcribed. They followed a topic guide used by all researchers flexibly and aimed to address the following ‘issue questions’ related to innovative service delivery [33]:

- What were the drivers for the localised practice change?
- What process was gone through to make the change? What were the barriers and facilitators to change?
- What impacts has this had on service delivery and for patients?
- If and how have you been able to sustain this service?
- What advice would you have for another service who may wish to adopt a similar practice change?

Participants were asked to discuss the specific area(s) of good practice identified via the survey response about their MAS, but were also given the opportunity to identify and discuss additional aspects of practice they felt were good or innovative in their service. In this way each case study was not limited to only including practices related to the area of innovation identified in the initial survey response.

For each case the interview transcripts were brought together to compile a detailed description of the case [35] including direct quotations. The initial case was written by the lead member for that site (CS/SS/LG or SB) and then all cases were also reviewed by the first or second author (CS or SS) alongside the interview transcripts to check accuracy and consistency of presentation across cases. Following this a cross case analysis [30] was conducted by the first author (SS) using deductive thematic analysis where thematic categories related to innovative approaches to addressing service challenges which also aligned to features of a person centred MAS pathway identified in the guide for innovative practice previously published by the authors [32]. The innovative practices were then compared across cases, with common and unique features identified and presented in this paper. Since the interviewees were not limited to only identifying the innovative practice first reported in the survey, each site features multiple elements of innovative practice that emerged from the data. The final themes were reviewed and agreed by the second author (CS). The full case studies are provided in a separate report [36].

## Results

Respondents to the survey came from England and Wales. There were 88 responses in total, although 39 were removed for incompletion or representing duplicate responses; 34 consented to take part in the survey but did not complete any substantive part of the survey and 5 responses were identified as duplicates.

Forty responses from England and nine from Wales were included in the analysis of the survey. Twenty-four participants agreed to be approached for participation in a case study. Following review of the site responses concerning innovative practice and consultation with the expert group, sixteen sites were approached to participate in the case studies. One site did not respond to follow up. Fifteen sites took part in case study data collection.

There was a broad geographical spread of respondents (see Table 1) with the greatest representation from the North East and Yorkshire region.

**Table 1:** Location of MAS

Area	ICS/Health Board	Number of responses	Mental Health/ Community/ Care Trust	Acute NHS Trust	Other
North East and Yorkshire (*n* = 15)	Humber, Coast and Vale	2	12	3	
	North East and North Cumbria	5			
	South Yorkshire and Bassetlaw	4			
	West Yorkshire and Harrogate	4			
North West (*n* = 8)	Cheshire and Merseyside	1	8		
	Greater Manchester	6			
	Lancashire and South Cumbria	1			
Midlands (*n* = 2)	Coventry and Warwickshire	1	2		
	Birmingham and Solihull	1			
East of England (*n* = 3)	Hertfordshire and West Essex	2	3		
	Norfolk and Waveney	1			
South West (*n* = 2)	Cornwall and the Isles of Scilly	1	2		
	Bristol, North Somerset and South Gloucestershire	1			
South East (*n* = 4)	Kent and Medway	2	3		1
	Sussex	2			
London (*n* = 6)	North Central London	2	6		
	North East London	1			
	South East London	2			
	South West London	1			
Wales (*n* = 9)	Aneurin Bevan	1	4	4	1
	Betsi Cadwaladr	2			
	Cardiff and Vale	2			
	Cwm Taf Morgannwg	1			
	Powys	2			
	Swansea Bay	1			
Total		49	40	7	2

### Service design

In England, the majority of the respondents (77%) were reporting for an individual MAS rather than on behalf of all MAS within their organisation (e.g. all MAS within a Trust). In Wales about half were reporting for an individual service (56%) and half for their organisation (44%) (see Table 2).

**Table 2:** Service design by locality

Region	Type of service		Reporting	
	Embedded	Stand-alone	All	Single
North East and Yorkshire	4	11	5	10
North West	4	4		8
Midlands	1	1	2	
East of England	3			3
South West		2	2	
South East	2	2		4
London	1	5		6
**England**	**15 (38%)**	**25 (62%)**	**9 (23%)**	**31 (77%)**
**Wales**	**5 (56%)**	**4 (44%)**	**4 (44%)**	**5 (56%)**

In England, 38% of the services reported were MAS that were embedded with Community Mental Health Teams, and in Wales this was 56%. There was a fairly even split between services with independent (England 50%, Wales 44%) and unified management structures (England 48% Wales 56%).

### Format of MAS service appointments

The respondents were asked the about the format of their assessments and consultation appointments, as they were currently (December 2021) being delivered in their service(s) by stating what percentages of appointments were being delivered using each listed modality.

**Fig. 1:**
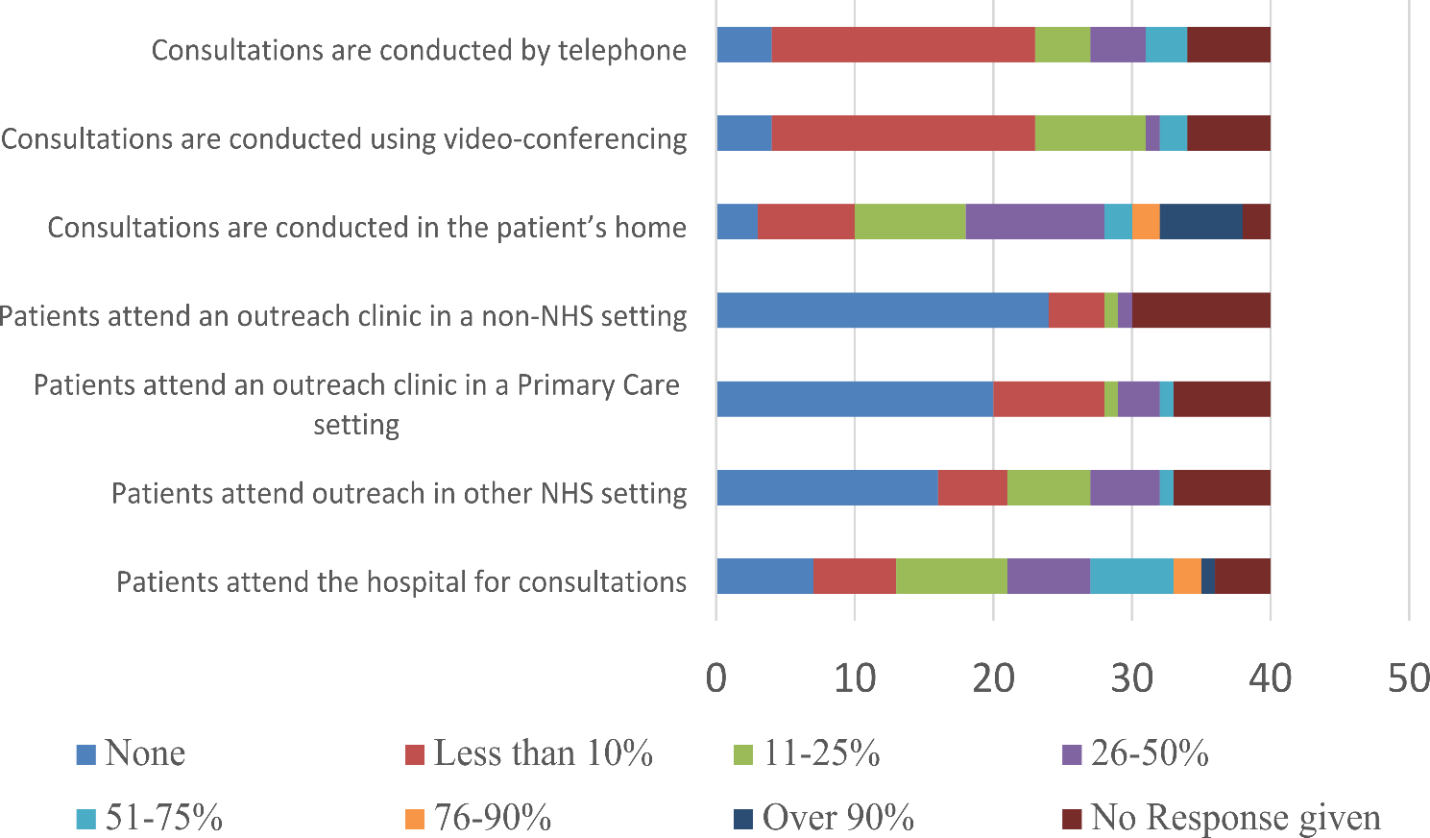
An overview of the way that appointments are delivered (December 2021)

Few services were offering appointments within outreach clinics such as primary care or non-NHS settings (Fig. 1). The most common modalities for appointments were consultation in the patient’s own home or attending the hospitals. Few services reported that appointments were delivered in a single modality (e.g., over 90% of services being delivered in one modality). Where this was the case, this tended to be using the patient’s own home. Where a mix of modalities were being used, these included telephone, video conferencing and hospital-based appointments as relatively common choices. Non-NHS settings and primary care were the least frequently used appointment settings.

### The impact of COVID-19 on modes of delivery

Participant responses to an open-text question about how appointments were delivered at the point of survey completion generally alluded to changes that had been made in response to the COVID pandemic. For most respondents the approaches that had been adopted in the peak of the COVID pandemic were still influencing practice, particularly with regards to adopting and retaining blended approaches to appointment mode.

*“The assessment is carried out face to face however, feedback of diagnosis has been delivered via telephone or video consultation.”*

“*Following the covid outbreak appointments are now and will continue to be offered, remotely via Microsoft teams, face-to-face at home or in clinic, or over the telephone. Where a telephone appointment is deemed appropriate and no other option available, outcomes are always discussed in the weekly multi-disciplinary team meeting to ensure enough information is available to go ahead with a diagnostic appointment or decide if further assessment is required at home. We have found the majority of our service users prefer to come to clinic for initial assessment.”*

Respondents were asked if the currently delivery model was different to their pre-COVID-19 model of delivery and if so how. In Wales, all respondents to this question indicated yes (*n* = 7), in England two thirds (*n* = 28/38) said the model was different.

Some respondents indicated how COVID had interrupted or delayed service changes that were already in the pipeline or being implemented.

*“Pre-COVID one part of the service was embedded in the CMHT. The decision had been made to make it a stand-alone service…but managed jointly with the already established MAS in the other half of the Trust which is commissioned by a different CCG [Clinical Commissioning Group]. A model had been agreed pre-COVID for the new service but this had to be re visited due to the situation.”*

*“Pre-COVID the service had began to implement a “Market Place” event, bringing together services/agencies who can offer a service to those living with dementia. This is something we plan to grow and develop as soon as .restrictions on service delivery are lifted.”*

Other respondents referred to the impact of COVID-19 on the rapid adoption of technology for remote consultations and blended approaches. This had streamlined services and offered more options to meet patient preferences. Although some respondents noted the increased use of technology was at the “cost’ of reducing face to face contact, which was not regarded as favourable.

*“We have now re-opened our clinic and are seeing most patients either in clinic or in home visit appointments, however, our team are now confident to offer remote and telephone assessments on rare occasions when it is needed.”*

In other services the impact of COVID was more temporary with modes of delivery returning to a pre-COVID models.

*“Staff have generally returned to their normal practice and preference which is to assess people in their home environment or within clinic."*

Respondents were asked if the current model reflected how they plan to deliver the service moving forwards. Most respondents in England (*n* = 30/38) and Wales (*n* = 5/6) indicated that this was how they plan to deliver the service moving forwards, although 21% of respondents in England said this was not the case.

Most respondents indicated that the elements of change they planned to retain related to offering optionality for patients.

*“We have found that family members and carers tend to prefer telephone consultations as they are more convenient. Home visits have also been found to improve access to the service for people with mobility issues or those who are concerned about attending hospital appointments. Looking at developing model that incorporates different consultation modes dependent upon need.”*

### Memory assessment service workforce

The survey asked which staff groups were represented within their MAS commissioned services (Table 3). All MAS based in Mental Health and Community Trusts included old age psychiatrists in their workforce, with a large majority also including registered mental health nurses, occupational therapists, and psychologists. Non-medical prescribers and healthcare assistants were also commonly employed staff groups.

**Table 3:** Which staff disciplines have dedicated sessional time in your MAS?

	^England^			^Wales^		
	^Mental Health/ Community/ Care Trust (34)^	^Acute NHS Trust (3)^	^Other (1)^	^Mental Health/ Community/ Care Trust (4)^	^Acute NHS Trust (2)^	^Other (1)^
^Old Age Psychiatrist^	^34^	^2^	^1^	^4^	^1^	^1^
^Neurologist^	^3^	^1^	^0^	^1^	^0^	^0^
^Geriatrician^	^0^	^0^	^0^	^0^	^1^	^0^
^Junior Doctor^	^20^	^2^	^0^	^0^	^0^	^0^
^Clinical Psychologist^	^29^	^1^	^0^	^3^	^1^	^1^
^Assistant Psychologist^	^18^	^1^	^0^	^1^	^1^	^0^
^Neuropsychologist^	^8^	^1^	^0^	^0^	^2^	^0^
^Occupational Therapist^	^27^	^2^	^1^	^4^	^2^	^1^
^Registered Nurse^	^9^	^0^	^1^	^1^	^1^	^0^
^Registered Mental Health Nurse^	^32^	^2^	^1^	^4^	^2^	^1^
^Advanced Nurse Practitioner^	^5^	^1^	^0^	^0^	^0^	^0^
^Non−Medical Prescriber^	^17^	^0^	^1^	^3^	^1^	^0^
^Dementia Navigator/Coordinator^	^7^	^0^	^1^	^3^	^2^	^0^
^General Practitioner^	^1^	^0^	^1^	^0^	^1^	^0^
^Healthcare Assistant^	^16^	^2^	^0^	^3^	^1^	^1^
^Other role^	^14a^	^1b^	^1c^	^1d^	^0^	^1e^

^a^Carer Support services, Alzheimer’s Society, Social Worker, GPwER, QAP, support worker, Admin, Alzheimers Society outreach worker, Administrator, Social Worker, Dementia Support Advisor, Social Worker, Admiral Nurses, Nurse Consultant, Social Workers, Occupational therapist assistant, Dementia advisor, Carer support worker

^b^Admiral nurses via Age Uk

^c^ GPwER

^d^Speech and language therapy

^e^Dementia advisor, Occupational therapy assistant practitioner

Some staff groups were less frequently represented, but still present in services, including neurologists and neuropsychologists, dementia navigators, and other staff groups such as Admiral nurses and Alzheimer’s society outreach workers.

We asked English respondents about the gaps or challenges in their current commissioned workforce model, with 78% of respondents identifying gaps. These fell into three broad categories: general capacity; the need for specialist staff; and staff to support post-diagnostic support.

First, some respondents indicated that staffing issues were related to general capacity.

*“Assistant psychologists are brilliant at doing neuropsychological assessments under supervision from a psychologist in another team - but are hard to retain- which means there are gaps when one goes and another is coming. Ideally we would benefit from a full time qualified psychologist but service is not commissioned or funded to include this.”*

*“No Occupational Therapist/s. No Assistant Practitioners (1 Fixed Term post at present), No Health Care Assistants/Support Workers. We do not have enough dedicated medic time in to the service. There are not enough staff in general.”*

Second, many respondents identified the need for staff and associated support pathways that can provide specialist or rarer dementia support, this included MCI, young onset dementia and less common forms of dementia.

*“Pathway for people who have a suspected alcohol-related brain damage and are still actively drinking*.”

Third, many respondents also identified needing more staff to enhance post-diagnostic support.

*“We would benefit from additional nurses and dementia navigators for post diagnostic support as currently we cannot meet the need.”*

### Commissioned services in MAS

To obtain a comprehensive overview of the MAS represented in our survey we requested respondents indicate which services they were commissioned to offer and deliver and where services were delivered locally but outside of the MAS (Table 4). Most MAS were commissioned to offer assessment and diagnosis to those over 65, with the majority also offering a diagnostic service to those under 65. Neuropsychology assessment, occupational therapy and cognitive stimulation therapy were also offered by most services.

**Table 4:** Overview of services that the Memory Assessment Service offer

Combined England and Wales	Commissioned as part of MAS	Available via the MAS but provided by another part of the NHS	Available via the MAS but delivered by a VCSE or other non-NHS partner	Unsure if available	Not available	Not reported
Memory assessment and dementia diagnoses for those aged 65+	42	1	0	1	0	5
Memory assessment and dementia diagnoses for younger people with dementia (under 65)	37	4	0	1	1	6
Neuropsychology assessment and input	35	6	0	1	1	6
Occupational Therapy assessment and input	34	4	0	1	4	6
Brain scanning (CT)	14	25	2	1	1	6
Brain scanning (MRI)	12	26	2	2	1	6
Brain scanning (PET)	10	26	1	2	3	7
Lumbar punctures for amyloid	3	13	1	7	15	10
Cognitive stimulation therapy (CST)	28	4	3	3	4	7
Dementia advisor/care co-ordinator	16	2	19	1	3	8
Dementia information sessions	22	2	13	1	4	7
Carer support	24	2	16	0	1	6
Advance care planning	22	1	7	1	9	9
Admiral nurse(s)	6	3	8	4	19	9
Other	9^f^	0	0	0	15^g^	25

^f^ 12 week post diagnostic review and care plan, START, Brainfood, Dementia clinical nurse specialists, Assistive technology occupational therapist, post diagnostic support, social workers, Medication and Care Planning, 3rd sector support agencies

^g^ Neuropsychology assessment

Where services referred to specialist components of assessment or diagnoses, such as imaging or biomedical assessment, these were less frequently commissioned as part of the service but could be accessed. Similarly, services that related to post diagnostic support (outside of CST) were less likely to be commissioned, but in most cases could be accessed, for examples information sessions or care co-ordinators. Specialist support, such as Admiral nurses, was not commonly offered or accessible elsewhere.

A follow-on question was asked (to respondents from England) about gaps in respondent’s current service commissioning. Of the 38 respondents to this question, 45% said they had gaps in the way the service is commissioned, 29% said there were no gaps and 26% were unsure. We asked respondents to provide further detail with regards to the gaps in service commissioning. Their responses fell broadly into two categories; support for MCI and less common forms of dementia and post diagnostic support and carer support.

*“Inadequate under 65s services, especially for non-neurodegenerative dementias (i.e. vascular and alcohol related brain injury).”*

*“Longer term follow-up is an issue. Currently*,* stable patients are discharged to the GP after 6 months*,* with referral back to the MAS when necessary. There are plans to increase support (e.g. Admiral Nurses) in the near future.”*

Finally, we asked English respondents whether they had plans in place for quality improvement over the coming 12-months. Most respondents (78%) indicated that they did, with 22% saying no or maybe. For the respondents indicating yes, the descriptions of their quality improvement plans broadly fell under three themes; streamlining and workforce recruitment (e.g. “we have trained our support workers to do phlebotomy and ECG’s so that we can complete all outstanding investigations at the first appointment”), introducing new services (e.g. “new business case to support MCI services”) and remodelling existing pathways (e.g. “Developing of Pathway Lead structure, reviewing the current pathway and designing a virtual diagnostic clinic for the county to help streamline assessments”).

### Multiple case study findings

A total of 44 staff and service users were interviewed across the 15 sites. Table 5 provides an overview of the case study sites including the key service features at each site. The interviews were conducted with eight Memory Service Mangers, five Consultants/Old Age Psychiatrists, seven carers and people with dementia, five Nurses, five Occupational Therapists, four Memory Support Workers, two Assistant Psychologists, four Clinical psychologists, three Assistant Practitioners and one GP Specialist. More granular details of the roles of staff interviewed have not been provided since this could lead to identification of participants where there is only one individual working in a particular role within a MAS. Examples of practice under each service feature from the case studies in the series are presented after the table.

**Table 5:** Case study participants and key service features

Case description	Location	Site description	Participant description	Service Features
Site 1: Improving the diagnostic process by streamlining multidisciplinary team meetings and medical input working with local external services (GP & audiology)	England	South of England in large inner city Stand alone service within a MH/community/care trust	4 staff interviews, 1 service user	Personalised care pathways Equity of Access Innovative/examples working with external services
Site 2: A team with specialist staff input commissioned to provide support from referral to end of life	England	South of England in large inner city Stand alone service within a MH/community/care trust	5 staff interviews 2 service users	Post diagnostic support Personalised care pathways Specialist staff (e.g. occupational therapy/admiral nurses)
Site 3: Providing tailored post-diagnostic support during the pandemic and beyond through adapted delivery of Cognitive Stimulation Therapy (CST) and other interventions	England	South of England in large inner city Stand alone service within a MH/community/care trust	3 staff interviews, 1 service user	Post diagnostic support Equity of Access
Site 4: Memory Hub and Younger Persons Memory Service with extended post diagnostic support	England	North of England Embedded service within an acute trust	3 staff interviews, 1 service user	Post diagnostic support Support for MCI and rarer dementia
Site 5: A psychology orientated assessment and diagnostic service with personalised pathways	England	Midlands Stand alone service within a MH/community/care trust	1 staff interview	Personalised care pathways Specialist staff (e.g. occupational therapy/admiral nurses)
Site 6: Rapid referral triage, specialist diagnostic pathways, and in-house health testing with CST	England	South of England Embedded service within a MH/community/care trust	3 staff interviews	Post diagnostic support Personalised care pathways Innovative/examples working with external services Support for MCI and rarer dementia
Site 7: Providing culturally tailored assessment and diagnosis	England	North of England Embedded service within a MH/community/care trust	2 staff interviews	Personalised care pathways Equity of Access Specialist staff (e.g. occupational therapy/admiral nurses)
Site 8: An approach to reducing assessment and diagnosis waiting lists with integration with primary care	England	North of England Stand alone service within a MH/community/care trust	1 staff interview	Innovative/examples working with external services
Site 9: Research Active Memory Assessment Service with a One Stop Shop and Innovative Brain Health Assessment and Support	England	North of England Embedded service within a MH/community/care trust	2 staff interviews	Support for MCI and rarer dementia
Site 10: Ensuring culturally appropriate assessment and diagnosis for people from the Somali community and at least 12-months of post-diagnostic support within the service	England	North of England Stand alone service within a MH/community/care trust	1 staff interview	Equity of Access
Site 11: Post-diagnostic support for people with dementia delivered by people with dementia and Promoting brain health in the local community	England	North of England Stand alone service within a MH/community/care trust	2 staff interviews	Post diagnostic support Location of memory services (e.g. Primary Care, community)
Site 12: Standalone MAS with specialist post-diagnostic primary care support	England	South of England Stand alone service within a MH/community/care trust	2 staff interviews	Post diagnostic support Innovative/examples working with external services Location of memory services (e.g. Primary Care, community)
Site 13: Nurse-led Memory Assessment Service located in primary care	England	South of England Stand alone service, social enterprise/not-for profit	Group interview with 3 staff 1 staff interview	Innovative/examples working with external services Location of memory services (e.g. Primary Care, community)
Site 14: MAS service with Specialist Occupational Therapy support	Wales	South Wales covering large cities and rural areas Stand alone specialist commissioned service	3 staff interviews 1 service user	Post diagnostic support Specialist staff (e.g. occupational therapy/admiral nurses) Innovative/examples working with external services
Site 15: Rural Service, nurse led, personalised approach	Wales	Central Wales in rural area Embedded service in acute trust	1 staff interview 1 service user	Location of memory services (e.g. Primary Care, community)

### Post diagnostic support

Half of the case studies featured innovative approaches to post diagnostic support. In some cases, this referred to specific interventions such as cognitive stimulation therapy. For example, at site 6, due to COVID staff felt alternatives to face-to-face group CST were needed to prevent people newly diagnosed with dementia from becoming isolated and under-stimulated. They developed options for delivering CST to people in their own home by way of a cross-borough project set up by the MAS psychologists and working collaboratively with psychologists from another MAS service within the same Trust.

*“[We were] very aware that*,* as the pandemic moved on and a lot of things had moved to online*,* that it was very much an issue around exclusion*,* digital exclusion*,* with older people predominantly. Maybe*,* many people within that group may not have… be able to access it. So*,* we are very mindful of that and the additional challenge of people with dementia as well. But we thought it would be worth giving it a go and seeing… and starting to develop a way of having conversations with people who were waiting for CST. About the idea and thinking about what support they would need to access the group online. And talking*,* also*,* to people who supported them*,* their families and carers*,* to see whether we could work together to enable them to access the group if they were interested.” (Site 6 - Staff member two).*

In other cases, innovative post diagnostic support meant adopting an extended model of post diagnostic support such as at case study site two where post diagnostic support is offered until end of life. At site two the lifetime support includes a one-month post-diagnostic review, followed by six-monthly comprehensive reviews which can be increased in frequency where complexities exist/arise, until the person with dementia has stabilised. Reviews are undertaken by either an Assistant Practitioner or a nurse dependent on the complexity of needs. At least one review annually is conducted in the person’s own home. The team includes occupational therapy and psychology staff who support diagnosis and post-diagnostic support. A lead practitioner and non-medical prescriber provide more intensive support for those with the most complex needs.

*“It was the beginning of a relationship and that for us was critical*,* that it’s not about being dumped with something and go away and sort out your affairs. … It’s about*,* yes*,* this is something we’re going to support you through and we will be following it up and you will be seeing [name] on a regular basis. So I think that’s … absolutely critical. You need continuity and you need somebody that you can begin to build trust with because it’s such an unknown territory at the beginning and you know it’s pretty terrifying because you think. Where do I begin?” (Wife of someone diagnosed with dementia by the service – site two)*

As in the above example, many of the reported cases rely on MDT approaches. For example, in site 12 a post-diagnosis support service within primary care was commissioned and is led by two GPs with a special interest in dementia. Both the memory assessment and post-diagnostic services work in collaboration with the Alzheimer’s Society.

### Personalised care pathways

In several sites, personalised care pathways meant operating a tailored approach to referral or triage systems (site one, five, six). For example, site six provides telephone triage for anyone referred into the service within 24-hours. This operates seven-days a week. The information gathered at triage alongside the referral is used to determine whether a person is assessed via the Tier One routine pathway, or Tier Two pathway for more complex cases. Tier Two includes neuropsychological assessment and MDT review. This triage is available 7-days a week as referrals dealt with by the nurses cover both memory assessment and functional mental health problems.

*“If we find that we identify risks within that conversation*,* where people need support within 24 h*,* or even a couple of hours*,* depending on what it is*,* we are able to do that. And I think that’s really important.” (Site 6 - staff member 3)*

Similarly, a site five, the service offers different pathways that are based upon patient needs and presentation at screening.

“*Clients we would sort of divide people up into different pathways depending on how they present. So from the most not for the most straightforward*,* the most kind of obvious. You know people that look like they’ve gotten established cognitive impairment or established dementia*,* but nobody has formally diagnosed them through to people that you may have comorbid major mental health difficulties may be considerably younger and may still be working. So we’ve we developed a number of different pathways.” (Site 5 - Staff member one)*.

The idea of the pathways ensures that people are not over or under assessed whilst ensuring that diagnostic accuracy is maintained.

*“So it’s making sure that we’re giving the right type of assessment to the to the right*,* to the right clients so that the with the exception of the formulation pathway*,* the other pathways people get the same. Assessment process. But how much of which bits they get will depend on how much they need.”(Site 5 - Staff member one)*.

### Support for MCI and rarer dementia

This was identified as a key theme present in two cases. Site four offers pre diagnostic support, diagnosis and post diagnostic support for younger people with dementia for as long as required.

*“I work with the younger person’s memory service and if someone needs nursing input prior to diagnosis*,* that will be through the younger person’s memory service*,* which is one of the reasons why there’s not dementia name in the service because a lot of people referred to the service won’t have dementia… And then when someone receives a diagnosis with dementia*,* then will remain within the younger person’s memory service until they have no need for us or until they’re 65” (Site 4 - Staff member 3)*.

This has also afforded the opportunity for re(de)diagnosis.

*“there was a kind of natural point there to start looking at diagnoses again when you repeat cognitive tests. And then you look back and think*,* well*,* right over a period of years*,* this hasn’t declined. This isn’t really consistent with your diagnosis anymore…. I think that is quite unique to our service because we do follow people up long term even if there aren’t major issues.” (Site 4 – staff member 3)*.

This was only possible because of the length of time people are followed up and has enabled staff to identify people who they may have expected to decline but have not. This approach may not be viable in services that do not offer an extensive follow up in this age group.

Site nine had developed an innovative service improvement project for people presenting to services unlikely to meet the threshold for dementia (e.g. MCI or similar). In this project a consultant intercepted and reviewed referrals to the regional MAS, redirecting those likely to present as MCI or functional impairment to a brain health clinic.

*“It’s not a perfect system*,* people … who are MCI*,* stage*,* still get through to the other consultants and people who have dementia stage illnesses are still getting through to me… over the last three months (the clinic) taking about 20% of the referrals which works out*,* if you do it retrospectively*,* works out at about the rate at which MCI is diagnosed amongst all referrals.” (Site 9 – staff member 1)*.

After a comprehensive assessment within the brain health clinic, clients are offered feedback and follow up care based upon their individual need. The idea of the clinic is to address the gap in support that people with subjective cognitive impairment might experience, and recognise that tailored support can be offered with beneficial outcomes for this heterogeneous group.

Site six operates a specific MCI post diagnostic pathway using funding provided by the Clinical Commissioning Group (CCG). This includes six group sessions that cover biopsychosocial advice to support the person to lead a healthy lifestyle and a workbook which provides advice and information related to the session content. People can receive the workbook even if they don’t want to attend the group sessions.

*“[The workbook includes] links to local resources that they can tap into so local exercise classes*,* …different voluntary organisations etc. People have found that useful*,* and … reiterates healthy living*,* health promotion*,* exercise*,* and … we … if someone wanted to give up smoking we can sign post them to smoking cessation or alcohol reduction teams. So we feel like we’re giving them something useful” (Site six – staff member 1)*.

The team have also been able to create a specific MCI register to ensure recall happens in a timely manner. This includes a six-monthly triage for those who are at high risk of conversion to dementia. If there has been a change the patient will come back in for assessment sooner.

### Equity of access

Examples of services that promoted equity of access included those that had adopted culturally appropriate practices. Site 7 was located in a city region with a high South Asian population. They noticed that members of this community were not being referred into the service at the same rate as white British people. When they did attend assessment, members of the South Asian community reported feeling the process was not culturally sensitive and staff reported difficulties administering the Addenbrookes cognitive assessment tool due to issues with language and the euro-centric nature of some of the questions. The service has worked with local GPs to dispel myths about dementia and to highlight the potential benefits of referring for/seeking a diagnosis. They undertook a project to develop a culturally appropriate version of the Addenbrookes which included revised questions and translation into Urdu and Hindi, and employ staff who speak some of the languages spoken in the local community so assessments can be conducted in their preferred language.

*“I’d experienced that challenge as a practitioner when I was delivering the original form of the Addenbrookes. Even interpreters were saying*,* well*,* that that’s not a fair tool. You’re not giving this this person a fair chance to*,* to demonstrate their cognitive abilities. That often resulted in fairly uncomfortable sessions where the interpreter was attempting to help out the individual who was being assessed and … trying to rephrase.” (Site 7 – staff member 1)*

### Innovative/examples working with external services

Site 13 had worked with external services to develop a rapid scanning pathway. Unlike other MAS’ who need to refer service users to local hospitals with long wait times for brain scans, the service had an innovative and bold scanning provision with very short waiting times. Both MRI and CT scans can be booked at the same time as the assessment appointment. The team also commissioned another more local scanning facility with longer wait times. This second service is more accessible by public transport and the more protracted wait times consider that not everyone will want a rapid service.

*“Look at your scanning pathway and see if there’s a possibility of doing that better as a kind of quick fairly*,* I mean*,* I’d say quick*,* easy win. I don’t do the contracts [S1] knows about that stuff*,* but you know*,* it seems to me to be a common theme of and a source of frustration or you know*,* some services get around that by requesting that the GPs get the scans done before they come into the service”. (Site 13 – staff member 3)*

A further example of novel approaches to external working was seen in Site 14 in which the team established a relationship with Digital Technologies Wales and was able to loan equipment, for example, iPads and Echo Dots, to trial with their service users.

*“How technology can advance somebody’s independence*,* looking at different ways in which we can enable people to be independent rather than just looking at kind of not using technology…there’s a perception there with older people*,* they’ll be less likely to use technology*,* but our outcomes of that are very different.” (Site 14 – staff member 4)*

The team based their interventions around technology that is affordable and is often already in people’s homes, or that can be easily purchased from websites such as Amazon.

### Specialist staff

Site 14 provided an example of a service that utilises specialist staff for a large portion of their service provision. The MAS occupational therapy (OT) service is a new service that delivers evidence-based pre-diagnostic assessment and post-diagnostic intervention. Each case is discussed at weekly MDT meeting and service users are referred to the OT team on a needs-based basis. For service users in receipt of OT support, once the OT team have completed their intervention (on average 12 weeks but can be shorter or longer) the service user will be discharged but will remain on the wider MAS caseload. To assist other healthcare professionals (e.g. GP), the OT team conduct re-assessments to determine if there have been any changes to an individual’s clinical or functional profile.

### Location of memory services

The location of services featured in some of the cases, often alongside innovative workforce practices or approaches, as a means to increase diagnosis rates and/or the efficacy of post diagnostic support. For example, in site 13 an innovative MAS nurse-led model was implemented. The service was reformed into a community service, operating out of primary care locations and delivering home visits.

In site 11 the service had identified some localities across the area it serves which have low referral and dementia diagnosis rates. They considered ways they could be targeted to increase referrals from these areas. Public brain health sessions were suggested as a possible solution, and these were commissioned for delivery by a local charity.

*“And the idea of the brain health sessions was to get down to grassroots. So we want [sessions] in supermarkets. We want them in libraries*,* in leisure centres. We were sort of saying*,* right*,* how do we get right down to … a really basic level with people who are out shopping and*,* oh*,* let’s talk about brain health. This is why it is important.” (Site 11 – staff member 1)*

The intention of the sessions is to increase availability and accessibility of support, raise awareness of dementia and its symptoms, reduce stigma by getting people talking and promoting the importance of getting a timely diagnosis.

## Discussion

This evaluation provides an overview of MAS in England and Wales demonstrating both common challenges and examples of innovative and unique service delivery. It showed that service variation exists which creates opportunities for services to tailor their provision to better meet local needs and improve patient outcomes. The case studies provide examples of practice for national commissioners as was as contributing to the evidence base for developing services, including those in LMIC. Examples of good and innovative practice often involved undertaking multiple service innovations, related to features of service provision (e.g. post diagnostic support, counselling), service structure (location of services, care pathways, accessibility), service workforce (use of GPs and specialists), and remit (supporting younger people, people with MCI or rarer dementia).

Services included in the case series strengthen the case for individual MAS to routinely (re)evaluate the design and delivery of their services. Many enforced changes to service provision due to COVID-19 generated opportunities to for services to streamline and refine MAS pathways, as identified by the services that took part in the survey. A key characteristic of the collective case studies was the importance of developing services that are responsive to local need and informed by evidence of such. Implementation science and practice change theory, such as the i-PARIHS model in the healthcare domain [37], draw heavily on the need for consultation in identifying service need and implementing local/inner context solutions. The importance of co design and consultation is also reflected in NHS commissioning guidance [38]. In our sites there was clear evidence of establishing local need and working collaboratively with staff or service users to inform solutions. For example, at site 6 solutions for the delivery of remote CST were driven by consultation with people with dementia and their families on the waiting list to receive CST. In our case studies, sites also spoke to the importance of commissioning support for the success of their approach – with top down organisational support representing key feature of practice change success.

That being said, our evaluation was also indicative of the challenges of local commissioning. Significant gaps and shortfalls identified by the survey (for example with regards to postdiagnostic support) indicate that local commissioning arrangements can also create uncertainty within dementia diagnostic and post-diagnostic support pathways. Whilst commissioning for local needs is undoubtedly important, it can lead to commissioning for the lowest cost resulting in disjointed service provision with gaps that may fall between the responsibilities of individual providers.

As an example, our survey findings indicate that despite existing evidence-based guidelines on dementia diagnosis and post-diagnostic support (e.g. NICE guidelines [8]), in some areas of the UK recommended services such as CST are reported to not be routinely available. There are a number of reasons why this might be the case including commissioners not providing funding for post diagnostic support at all, for specific CST services, or a lack of resources within commissioned services to deliver all aspects of a service they are commissioned to deliver. The latter could result from a need to prioritise resources elsewhere in the system to meet more pressing demands (e.g. to diagnosis post pandemic vs. post-diagnostic support), lack of staff/staff with the requisite skills to deliver particular interventions [21], or a perception that other services/interventions better meet local needs and so reflect a better investment of limited resources [38].

NHS commissioning guidance for dementia requires that commissioners take an approach aimed at reducing health inequalities [10, 38]. This involves identifying and meeting local need, such as demonstrated in sites adopting culturally appropriate assessment practices, but balancing this against “reducing unwarranted local variation in process and outcomes” [38], Overall, the commissioners’ priority is one that prioritises personalisation over place based approaches such that “the commissioning landscape…is characterised as much by the ability to personalise and shape care and support in response to individual needs and preferences, as it is by place-based approaches and new models of care” [39]. A third of the case series reflected the service feature of personalisation, with all cases reflecting an element of personalisation or person-centred approaches, potentially indicating the success of this commissioning principle. Although, given the survey format and complexity of commissioning arrangements and decision-making within local healthcare systems, it was not possible for us to have a more in-depth understanding of this issue and it should be a topic for future research.

In a few sites, such as site 9 that presented a Brain Health Service, the innovation had not yet met the threshold for commissioning, but nonetheless demonstrated an innovative local solution. Innovation at this site relied on individual motivation and skills to demonstrate solution, and whilst not (yet) showcasing sustainable service change, made a key contribution to the evidence base for future memory services. The brain health site reflects contemporary thinking that Brain Health Clinics are the next step in dementia care provision [12] and provides a working example of this in practice. The European Task Force on brain health services suggested the deployment of a second generation of memory clinics designed to offer evidence – based prevention in at risk individuals [12]. These clinics – conceptualised as Brain Health Services - will offer risk assessment (biomarkers and brain pathology), risk communication and personalised prevention. These are likely to become more important as earlier minimally or non-invasive dementia screening and diagnostic tests become more widely available (e.g. blood tests) and new disease modifying drugs come onto the market. These may lead to earlier presentation at MAS and a need to combine prescriptions of drugs where warranted, with a range of health advice on personalised prevention and risk reduction and ongoing monitoring.

However, personalised prevention is likely to be based on multidomain interventions [40] – and will likely put further pressure on services already struggling to offer PDS for dementia. Therefore, understanding the prevalence and exploring the reasons for existing shortfalls in this provision is an increasing priority. Service pressures will also arise from new treatments that require etiology to be clearly established (e.g. monoclonal antibody treatments) [41] in order to offer personalised treatment plans.

Examples from our collective case studies that address shortfalls in postdiagnostic care will be useful for national commissioning as well as provide examples for international service development. For example, two case study sites focused on meeting the needs of ethnically diverse communities, contributing evidence that may have additional utility for service development in LMIC. In the UK we are facing a projected seven-fold increase in dementia over the next five years in people from Global Majority communities compared to a two-fold increase in White British counterparts [42]. In the UK older South Asian adults are less likely to access dementia diagnostic services in a timely way, compared to rates for older white British adults [43]. Our case studies explored ways in which the diagnostic process can be adopted to be more sensitive and accurate as well as ways to reach into communities who may be reluctant to engage with services. In line with previous studies that suggest that simply scaling up services that work well in HIC will not work in LMIC [15], our findings indicate the importance of adapting services according to population characterises in order to achieve successful service outcomes. Whilst our communities exist in a HIC, there may be elements of learning that can be applied to the development of services in LMIC in South Asia and Somalia.

Previous research in HIC with similar models of care have indicated that patient experience can be adversely affected by the articulation between the MAS and other services, as in our evaluation, a study in Australia patients report significant gaps in post diagnostic support available to them [13], which was attributed to the articulation of services where GP discharge occurs. Our case studies also reflected the importance of the physical location of services or the points of contacts within the service pathway. Services that improved relationship with GPs or ancillary services elicited better patient outcomes. The importance of improved articulation existed at both ends of the pathway, referral and discharge. For example, in one of our cases (site 11) – the service point of contact was early in the pathway (pre diagnosis) and located in the community. This service was attempting to address the issue of low local diagnostic rates by running brain health sessions within the local community.

Innovation relied upon the creative use of staff as well as location. In our sites a range of staff have been shown to be equipped to reliably obtain accurate diagnosis. This is in line with published finings such as previously evaluated service addressed workforce issues by implementing advanced nurse practitioner (ANP) roles across four localities of a large NHS Trust. The ANP were upskilled and able to meet competencies related to assessment and diagnosis of dementia, thus reducing reliance on medical staff within the MAS [44]. In another evaluation structured assessment by AHP’s was shown to be just as accurate as a formal MDT judgment [45]. The use of community nurses at the point of screening has also been shown to have potential to enhance the speed and accuracy of dementia pathways [46]. Taken together, these findings support the recommendations of the WHO which advocated the use of health care workers in the community to increase diagnosis rates in LMIC. However, simply scaling up interventions that have worked in HIC is unlikely to generate optimal outcomes [14].

### Limitations

This evaluation was limited by the number of services that the responded to the survey in the first instance (*n* = 49) which served as the sample from which the practice cases were obtained. However, given our evaluation was designed to seek examples of good or innovative practice, our self-selecting sample was primed to be receptive to requests for information about their services and provide access to their services for the subsequent case study component of the evaluation. A further limitation was that in conducting the case studies it wasn’t possible to obtain service user experience in all cases, so in most cases the staff perspective dominated. Previous research has suffered the same limitation in demonstrating that the structural and process characteristics of MAS in England are unrelated to HRQOL [47]. However the service innovations and structural and process characteristics may positively or adversely affect patient experience, and this should be further investigated via through qualitative exploration.

Whilst we did not record details of MAS accretion with MSNAP in our survey or in the case studies, it may be considered a limitation of the study that not all MAS taking part in the cases were accredited. Some were accredited, some were waiting to be and others reported being limited applying for accreditation because of the way that they were commissioned. However, previous research shows that MAS who had been accredited by MNSAP did not stand out as different in regard to structural or process characteristics to those that had not been [21].

A further limitation of the study is that we did not consider access to service as part of the pathway. Previous research has shown that the experience of accessing the service is as important for patient experience and outcomes as their experience of the service itself [13]. Future research should consider the local pathway in its entirety.

## Conclusion

Our findings, taken together with the available evidence, suggest a significant shortfall in postdiagnostic services. This is particularly important given new dementia disease modifying treatments in the pipeline and the proposed second generation of memory services that will extend assessment to individuals at risk of dementia.

Whilst incentivisation of diagnosis in the UK led to increased diagnosis this created the “gap” of postdiagnosis service provision that has not been claimed by a specified service. Our collective case studies series offered solutions to the PDS shortfall. For example, the offer of CST remotely, with the benefit that it is not subject to the limitation of rural or remote geography – ideal for implementation in many LMIC, that may rely on digitation and remote delivery of services [14].

Our findings indicate that services should reflect local need. Population characteristics may be a important drivers for successful service outcomes – and providing a variety of services as examples that work well with specific communities is useful. Furthermore,

MAS will need evolve to keep up with our conceptualisation of cognitive impairments and the treatments on offer, which are increasingly becoming both more personalised (based on etiology) and preventative. Services need to prepare to deliver a changed offer and to meet national clinical guidelines on expected service provision and standards of pre-, diagnostic and post-diagnostic care for people with dementia. This will entail working collaboratively with stakeholders and commissioners, working across the dementia pathway, and balancing non-standard models of working with equitability. Forums which showcase good practice, that often remain unseen, will be a useful resource for developing national and international memory assessment and support services.

## Data Availability

Data may be made available from the authors upon reasonable request by contact Dr Sarah J Smith at s.j.smith@leedsbeckett.ac.uk.
